# An Uncommon Presentation of Paraneoplastic Leukemoid Reaction (PLR) in a Rare Case of Adenosquamous Carcinoma (ASC) of the Gallbladder (GB): A Case Report

**DOI:** 10.7759/cureus.41040

**Published:** 2023-06-27

**Authors:** Muhammad Usman A Khan, Abdullah Shehryar, Muhammad Imran, Muhammad Bilal Ch., Ahmadullah Baig

**Affiliations:** 1 General Surgery, Allama Iqbal Medical College, Lahore, PAK; 2 Internal Medicine, Allama Iqbal Medical College, Lahore, PAK; 3 Pathology/Histopathology, Allama Iqbal Medical College, Lahore, PAK

**Keywords:** incidental gall bladder carcinoma, gi malignancies, cholecystectomy, paraneoplastic leukemoid reaction, gallbladder adenosquamous carcinoma

## Abstract

This case report describes an unusual paraneoplastic leukemoid response presenting in a rare instance of gallbladder (GB) adenosquamous carcinoma (ASC). Adenocarcinoma is the most prevalent histological subtype of GB carcinoma, which is most frequently diagnosed in people in their sixth and seventh decades of life. Adenosquamous and squamous variations are uncommon. Rarely have reports of paraneoplastic leukemoid reaction (PLR) in GB carcinoma been made; this reaction is characterized by a white cell count exceeding 50,000/mm^3^ in combination with solid malignancy. PLR has most commonly been found in association with lung carcinoma.

In this instance, a 40-year-old man presented with right upper abdominal pain and a total leukocyte count of 26 x 10^9^/L. The patient was initially treated on the lines of acute cholecystitis. But when the abdominal symptoms and leukocytosis did not settle, open cholecystectomy was performed. The results of the histopathological analysis showed that the GB had adenosquamous cancer. The white cell count increased even after surgery. Leukocytosis in the patient was looked into further to rule out hematological malignancy and other possible reasons. Sadly, the patient expired before any treatment could be started.

The cancer GB carcinoma is uncommon and aggressive. Despite its rarity, ASC should be included in the differential diagnosis. PLR is an unusual manifestation associated with GB carcinoma. A thorough investigation, including a complete blood count, can help identify this paraneoplastic syndrome in patients with elevated white cell counts.

## Introduction

Gallbladder cancer is a rare disease, often diagnosed at advanced stages. It accounts for two to four percent of all gastrointestinal malignancies with adenocarcinoma being the predominant histological type, accounting for 80%-90% of all GB malignancies. Squamous and adenosquamous carcinoma (SCC/ASC) are encountered rarely with incidence ranging from 1% to 12% [1]. Histologically, ASC has both glandular and squamous components. Squamous component ranges from 25% to 90% [2].

Though GB ASC is itself a rare entity, an even rarer association is a leukemoid reaction occurring with it. Paraneoplastic leukemoid reaction (PLR) is defined as a white cell count greater than 50,000 associated with solid malignancy [3]. In order to establish a leukemoid reaction, other causes of leukocytosis like infection, bone metastasis, or hematological malignancy should be ruled out [3-4]. PLR is found in a number of solid malignancies (e.g. lung, liver, pancreas), in around 10% of the cases [3], but its association with carcinoma GB is very rare. It is reported only a few times in the literature review [2]. We are able to find only four case reports of GB cancer presenting with a leukemoid reaction. Of these case reports, three cases of adenocarcinoma of GB are associated with leukemoid reaction. Only one case of ASC GB associated with leukemoid reaction has been previously reported. 

## Case presentation

A 40-year-old male with no significant past medical history presented to the outpatient department with a history of pain in the right hypochondrium (RHC) for the last 20 days. The patient had no history of fever, cigarette smoking, alcohol use, or any family history of cancer. On examination, he had tenderness in the RHC and Murphy’s sign was positive. However, the patient had no anemia or jaundice, and no mass was appreciated on the abdominal examination. Complete blood count (CBC) at presentation showed a total leukocyte count (TLC) of 26 x 109/L. The patient was admitted to the surgical ward on lines of acute cholecystitis.

On admission, ultrasonography was done which showed features of acute cholecystitis that had increased wall thickness of GB with multiple stones in the GB lumen. An enlarged lymph node at porta hepatis was also seen. All other investigations like liver function tests (LFTs), serum amylase, and lipase levels were unremarkable. Our initial plan was the medical management of the patient with antibiotics, IV fluids, and painkillers, followed by interval cholecystectomy.

However, the TLC count was continuously increasing with a slight improvement in abdominal pain. Considering the young age and male gender of the patient, differential diagnosis of GB cancer was put down in the list or not considered at all. Due to the static condition of the patient despite medical management, it was planned to proceed with cholecystectomy. An expected difficulty in surgery in settings of acute cholecystitis led us to decide in favor of open cholecystectomy through Kocher's incision rather than a laparoscopic approach. At surgery, multiple stones were present in the GB lumen. The wall thickness of GB was unusually increased, and it was firmly adherent to the liver bed. 

Post-operatively TLC count of the patient increased from 35 x 109/L on the first post-operative day to 42 x 109/L on the 12th post-operative day. On the same (12th post-operative) day, a report of the pathological analysis revealed pT2b N1Mx ASC of Gb, histologic grade II disease with lymphovascular and perineural invasion. Table 1 shows a rising trend of white cell count from the day of admission of the patient to the hospital.

**Table 1:** Hemogram showing upward trend of white cell count. PAD, post-admission day (after 1st admission); WBC, white blood cells; RBC, red blood cells; HCT, hematocrit; MCV, mean corpuscular volume; MCH, mean corpuscular hemoglobin; MCHC, mean corpuscular hemoglobin concentration

	25^th^ PAD	36^th^ PAD	42^nd^ PAD	43^rd^ PAD	44^th^ PAD	Normal values
WBC count	63.3	100.7	120.2	131	167.2	4-11 x 10^9^/L
Total RBC	3.69	3.68	3.87	3.53	3.34	3.8-5.2 x 10^12^/L
Hemoglobin	9.3	9.0	9.6	9.2	8.3	13-18 (g/dL)
HCT	28.8%	35.7%	-	29.1%	30%	35%-46%
MCV	78	97	-	82.4	89.8	77-95 fl
MCH	25.2	24.5	-	26.1	24.9	26-32 (pg)
MCHC	32.3	25.2	-	31.6	27.7	32-36 (g/dL)
Platelets	812	430	486	387	573	150-400 x 10^9^/L
Neutrophils	88.5%	91.2%	-	84%	-	40%-80%
Lymphocytes	3.7%	4.6%	-	4%	-	20%-40%
Monocytes	-	-	-	2%	-	2%-10%
Eosinophils	-	-	-	-	-	1%-6%

**Figure 1:**
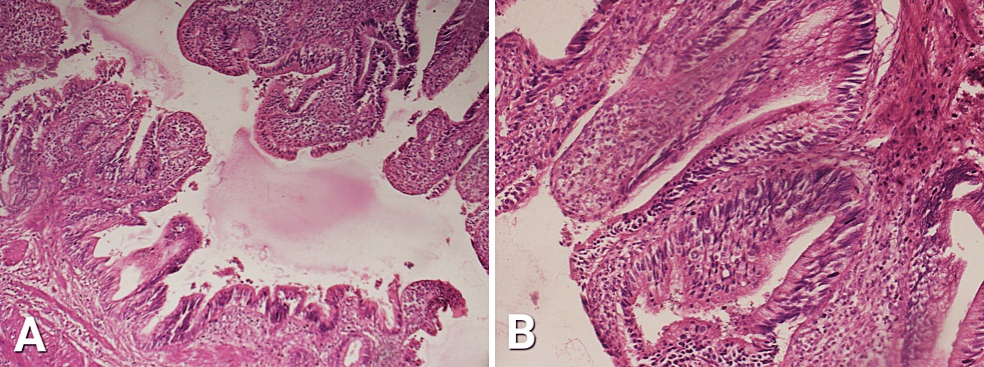
Wall of gallbladder with mucosal projections and Rokitensky's Aschoff sinuses. A: 10X magnification. B: 40X magnification

Detailed histopathological analysis showed a wall of GB with mucosal projections and Rokitensky’s Aschoff sinuses. Epithelium also showed focal dysplasia. This is shown in Figure 1.

**Figure 2:**
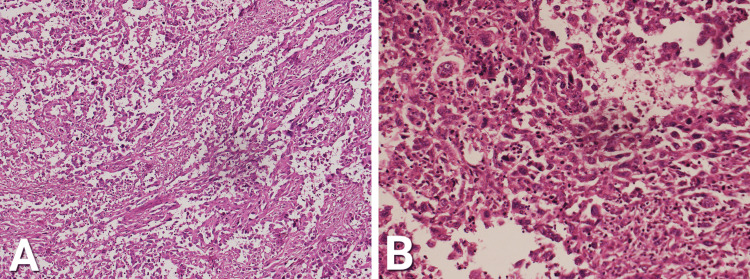
Wall of GB with sheets, vague glandular pattern, and individually scattered cells with marked nuclear pleomorphism. A: 10x magnification. B: 40x magnification GB, gallbladder

Moreover, the wall of GB was found infiltrated by malignant neoplasm-forming sheets, vague glandular patterns, and individually scattered cells with marked nuclear pleomorphism. There was evident desmoplasia in the surroundings. The whole constellation is shown in Figure 2.

**Figure 3:**
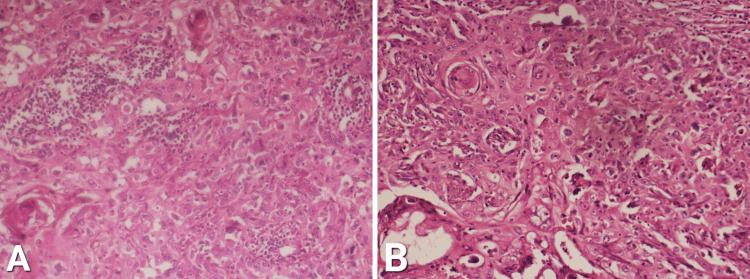
Areas of squamous differentiation with cells showing abundant cytoplasm and markedly pleomorphic nuclei. A: 10X magnification. B: 40X magnification

Areas of squamous differentiation with cells showing abundant cytoplasm with markedly pleomorphic nuclei are shown in Figure 3.

**Figure 4:**
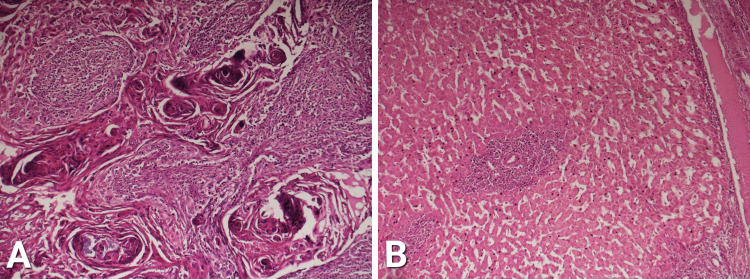
A: Areas of squamous differentiation with prominent keratin pearls. B: Portion of liver parenchyma adherent to gall bladder wall at porta hepatis, showing chronic inflammatory infiltrate.

Areas of squamous differentiation with cells showing abundant cytoplasm and markedly pleomorphic nuclei. Keratin pearls are also prominent. A portion of liver parenchyma adherent to the GB wall at porta hepatis shows chronic inflammatory infiltrate. Elaborated in Figure 4.

**Figure 5:**
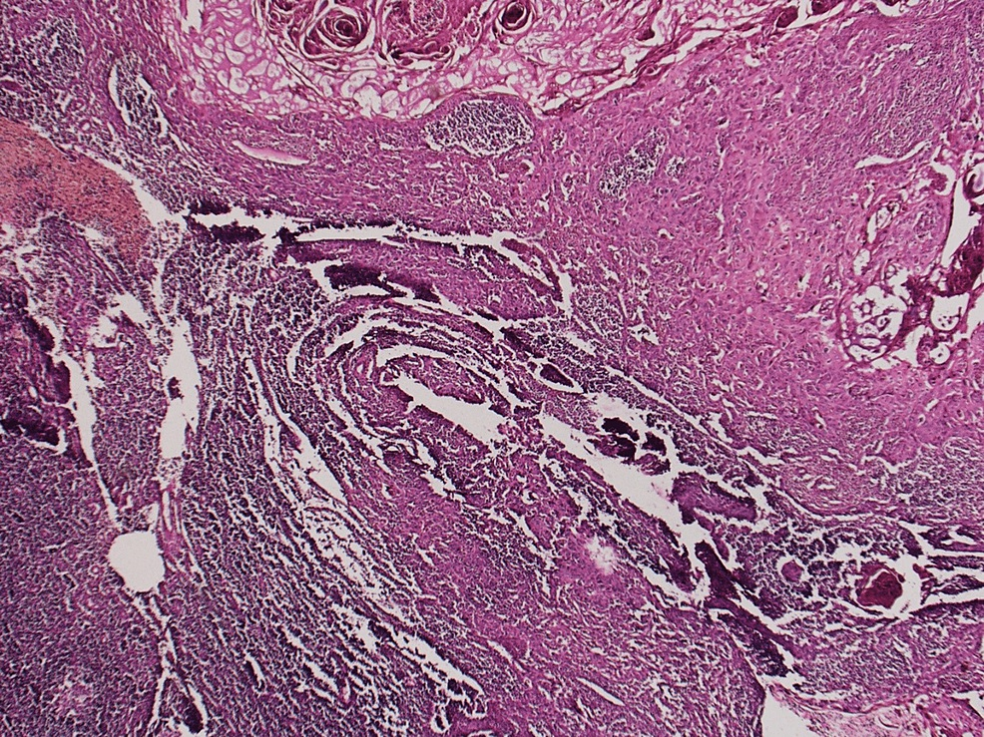
Lymph nodes from Calot's triangle show effacement of architecture, and replacement with malignant cells.

Lymph nodes from Calot’s triangle revealed effacement of the architecture, showing replacement with malignant neoplastic deposit. This is shown in Figure 5.

An infective screen including chest X-ray, wound check, urinalysis, and blood culture was normal. On the 15th day of open cholecystectomy, CT chest, abdomen, and pelvis were done for metastatic workup as well as to look for the cause of raised TLC. CT showed a recurrence of disease in the GB fossa and suspicion of collection in the RHC. Therefore, exploratory laparotomy through a midline incision was done to look for any abdominal collection to be the likely cause of raised TLC, but no abdominal collection was appreciated. However, a recurrence of the disease in GB fossa was seen. 

**Figure 6:**
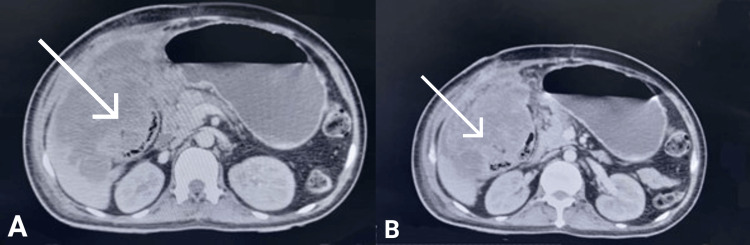
Heterogeneous mass can be seen infiltrating the liver, hepatic flexure of colon, and duodenum (arrows in A & B).

Now we had a patient whose TLC was not settling, but on the other hand, his adjuvant chemotherapy was being delayed. Once the patient recovered from the post-operative phase of exploratory laparotomy, he was referred to oncology for necessary further workup and chemotherapy. But he defaulted on treatment and again returned five days later in a surgical emergency with a complaint of bilious vomiting. Again, a CT scan was done on the 44th day of open cholecystectomy, which showed a heterogeneous mass measuring 12 cm x 8 cm x 15 cm infiltrating the liver, hepatic flexure of the colon, and the duodenum, as shown in Figure 6.

On the 10th day of re-admission, TLC was 195 x 109/L with 94% neutrophils with a peripheral smear showing toxic granulocytosis. After consultation with the hematology department, bone marrow biopsy followed by BCR-ABL genetic testing was in our plan to rule out blood malignancy. However, the condition of the patient deteriorated during the admission. He became increasingly cachexic, had a continuous fever, poor oral intake, and symptoms of abdominal fullness due to the mass effect. He expired before these tests could be performed. Thus, based on the results of peripheral smears alone, we conclude that the leukemoid reaction could have occurred as a paraneoplastic syndrome.

## Discussion

Gallbladder carcinoma is a rare malignancy with a poor prognosis, characterized by aggressive behavior and low survival rates. Risk factors include porcelain GB, female gender, obesity, older age, southeast Asian geographic group, choledochal cyst, bile duct abnormalities, GB polyp, primary sclerosing cholangitis, typhoid, and family history [1, 5-8]. The clinical presentation can vary from asymptomatic to overlapping features with cholecystitis like abdominal pain, tenderness, and leukocytosis [8]. Advanced disease may manifest with weight loss, anorexia, and jaundice.

The histological types of GB cancers are adenocarcinoma, squamous cell carcinoma/adenosquamous carcinoma (SCC/ASC), and very rare GB neuroendocrine tumors and carcinoids. The vast majority of GB cancers are adenocarcinomas, with squamous and adenosquamous varieties constituting a minority. There is limited published information on the SCC/ASC of the GB. In one study of 94 resected invasive GB cancers, squamous differentiation was identified in eight cases [1]. In this study, the mean age of presentation in the group of SCC/ASC was 57.8 years (range 50-70). The average age of patients with SCC/ASC in other studies was about 60 years [3]. In our case report, the age of the patient was 40 years. According to this study, all the cases with SCC/ASC presented in the advanced stage, classified as pT3 and higher or Stage III and higher [1]. This has been attributed to a high proliferative index of the squamous component in these tumors. These findings are consistent with the histopathological analysis of our study. Similar findings were reported in another study [3] that showed significantly higher percentages of T4 disease (61.8%) and N1 nodal involvement (58.8%) in SCC/ASC patients as compared to the ASC group.

Another significant feature of our case is a leukemoid reaction, which means TLC count greater than 50,000/mm3. It is found in many of solid malignancies, but its association with ASC of GB is rare. A study evaluated 758 patients with solid tumors and extreme leukocytosis [9]. Only one case of ASC GB associated with leukemoid reaction has been previously reported to the best of our knowledge [10]. The patients diagnosed with a PLR typically had neutrophil predominance [9]. The peripheral smear of our case also showed leukocytosis with similar findings. There are three proposed mechanisms for increased white cell count in cancer patients: extensive bone marrow metastases, necrosis of the tumor mass, and cytokine production by the tumor cell, such as colony-stimulating factor (G-CSF), which can promote bone marrow cell proliferation and differentiation [10]. Suzuki et al. [11] discovered increases in the levels of many cytokines in tumor culture supernatants, contrary to previous findings that stated a single dominating cytokine released by the tumor was in charge of the leukemoid response. The severity of the granulocytosis and the extent of tumor necrosis were not correlated by Kitamura et al. [12], but they were also unable to rule out the potential of significant tumor necrosis leading to granulocytosis [9].

While there is no specific management approach for leukemoid reactions associated with solid organ malignancies, early identification, prompt intervention, and addressing the underlying cause are crucial for improving outcomes. In this particular case, the patient's deteriorating condition and limited therapeutic options might have contributed to the unfortunate outcome.

In patients having malignant tumors with a white cell count of more than 50,000/mm3, we should think about PLR. A rapid diagnostic approach to identify the underlying cause and early administration of the most effective treatment can result in a better prognosis for such individuals [9]. In our particular case, we were planning to get a bone marrow biopsy followed by BCR-ABL genetic studies to rule out leukemia more confidently. But our patient expired before these tests could be performed. Nevertheless, based on the clinicopathologic examinations and the result of the peripheral smear, we can say with confidence that it was a leukemoid reaction. Extreme leukocytosis in the presence of cancer delivers a diagnostic conundrum. Still, we would opt to include the fact of not having bone marrow biopsy and BCR-ABL done, as a limitation of our study.

## Conclusions

In conclusion, GB SCC/ASC are rare. When they are diagnosed, they frequently have advanced stages (pT3 and pT4), which may be partially explained by the greater proliferation rate of the squamous component in ASC of the GB. The etiopathogenetic and molecular differences, if any, between this histologic type and typical adenocarcinomas require further research. Moreover, patients having solid malignancy with TLC count greater than 50,000/mm^3^ may have PLR. To see it in a reverse manner, for any patient who presents with RHC mass and TLC count raised beyond 50,000/mm^3^, GB malignancy can be considered. Thus, a simple investigation such as a complete blood count can help us to suspect a malignancy when more detailed imaging modalities are not readily available. The leukemoid reaction has no clear management guidelines in literature because of its rarity of presentation and is associated with poor prognosis and overall survival when associated with cancers.
